# Myxedema coma: four patients diagnosed at the Internal Medicine Department of the Dr. Negrin University Hospital in Spain

**DOI:** 10.11604/pamj.2019.34.7.19164

**Published:** 2019-09-03

**Authors:** Noel Lorenzo Villalba, Abrar-Ahmad Zulfiqar, Vanessa Saint-Mezard, Maria Belen Alonso Ortiz, Melek Kechida, Nuria Fuertes Zamorano, Saturnino Suárez Ortega

**Affiliations:** 1 Service de Médecine Interne, Diabète et Maladies Métaboliques, Hôpitaux Universitaires de Strasbourg, Strasbourg, France; 2 Service d’Endocrinologie, Centre Hospitalier Agen, France; 3 Servicio de Medicina Interna, Hospital Universitario de Gran Canaria Dr Negrin, Spain; 4 Service de Médecine Interne et Diabète, Hôpital Universitaire Fatouma Bourguiba, Tunisia

**Keywords:** Myxedema coma, hypothyroidism, hormone replacement therapy

## Abstract

Myxedema coma is a rare complication of hypothyroidism. Clinical examination may reveal hypotension, bradycardia, and hypothermia. Laboratory tests may show hyponatremia, lipid disorders, and elevations of creatine kinase, liver, and cardiac enzymes. We describe four cases diagnosed in our hospital during the period 1999-2017. The patients were related to amiodarone treatment, noncompliance with hormone replacement therapy, or a new diagnosis of hypothyroidism. Intravenous hormone replacement therapy was effective in three of the cases and one died. The outcome of this disease may be fatal as seen in one of our cases.

## Introduction

Myxedema coma is a rare condition and represents the extreme expression of severe hypothyroidism. It may occur in patients with a background of long-standing undiagnosed hypothyroidism or may be precipitated by an underlying medical or surgical condition. It may also result from prolonged absence of thyroid hormone replacement treatment in patients with known hypothyroidism [1]. It has rarely been linked to drugs such as lithium [2] or amiodarone [3]. Prolonged hormone replacement therapy discontinuation has been related to hypothyroidism coma, although this is uncommon given the length of time required without thyroid replacement for this syndrome to appear.

## Methods

We collected all the clinical and biological data from patients diagnosed of myxedema coma in internal medicine department of Dr Negrin University Hospital in the period December 1999 to February 2017. Four cases were diagnosed in the study period.

## Results

### Case one

A 86-year-old woman presented with ventricular extrasystoles one year prior to her current admission. She was started on amiodarone 400 mg five days per week. Her thyroid hormone levels were normal prior to initiation of amiodarone. Six months prior to admission, she started complaining of cold intolerance, with bradycardia and myxedema accompanied by occasional vomiting and a change in her mental status. Upon admission, her blood pressure was 170/80 mmHg, body temperature 32.6ºC, and heart rate 40 bpm. The physical examination revealed stupor, myxedema, loss of the outer third of the eyebrows, and macroglossia. Bilateral lower limb edema was noted on examination. The remainder of the examination was non-contributory. Laboratory tests showed T4 0.2 mcg/dL (normal values: 4.5-12.5), thyroid stimulating hormone (TSH) > 60 mUI/l (normal values: 0.4-4), sodium 127 mEq/l, creatine phosphokinase (CPK) 736 U/l, glutamic oxalacetic transaminase (TGO) 122 U/l, lactate dehydrogenase (LDH) 954 U/l, and cholesterol 382 mg/dl. Blood gas analysis showed pH 7.35, paO2 49 mmHg, and pCO2 67 mmHg. Abdominal X-rays were consistent with the presence of paralytic ileus, and the ECG showed sinus bradycardia (40 bpm), QT 0.68 seconds, low voltages, and diffuse disorders of repolarization. Treatment with IV thyroxine hormone (250 mcg), hydrocortisone, and ventilatory support were initiated. During the hospitalization the patient developed refractory hypotension, and she died on day 2 from cardiac arrest nonresponsive to resuscitative efforts.

### Case two

A 72-year-old woman was admitted to the emergency department for memory loss, behavioral disorders, and confusion for two weeks. Her medical history was positive for primary hypothyroidism treated with 100 mcg thyroxine daily which she had discontinued three months prior to admission. Upon arrival, her blood pressure was 150/90 mmHg, heart rate 50 bpm, and body temperature 35.1ºC. On physical examination the patient was confused and agitated. Myxedema, loss of the outer third of the eyebrows, and bilateral lower limb edema were noted on examination. Deep tendon reflexes were prolonged. Laboratory tests revealed T4 0.1 mcg/dL, TSH 80 mUI/l, and normal liver and renal function tests. White blood cell count, hemoglobin, and electrolytes were within the normal range. The patient received IV thyroxine and hydrocortisone with clinical and laboratory improvement.

### Case three

A 86-year-old woman was admitted to the emergency department for evaluation of altered mental status. Her medical history was positive for systolic hypertension, obesity, and dyslipidemia. One year prior to her present admission, the patient presented with weight gain, loss of appetite, and labile mood. During the two weeks prior to admission, she became progressively more non-verbal with occasional confusion and daytime sleepiness. On the day of her admission she was nearly unresponsive. Her blood pressure was 160/60 mmHg; heart rate was 45 bpm, and body temperature 34ºC. Physical exam revealed drowsiness, myxedema, dry skin, and bilateral lower limb edema. The remainder of the physical exam was unremarkable. Laboratory tests showed T4 0.1 mcg/dl, TSH 55 mU/ml, cholesterol 285 mg/dl, triglycerides 119 mg/dl, aspartate aminotransferase (AST) 71 UI/l, alanine aminotransferase (ALT) 20 UI/l, CPK 1374 UI/L, LDH 612 U/L, sodium 117 mEq/L, potassium 2.7 mEq/l, calcium 7.19 mg/dl, and phosphorus 2.6 mg/dl. Antinuclear and antithyroglobulin antibodies were negative, but antithyroid peroxidase was 1/160. Sinus bradycardia was noted on the ECG. The patient was given IV thyroxine and hydrocortisone. On the second day of admission, the patient was clinically improved, and her blood tests showed improvement of the thyroid functions.

### Case four

A 67-year-old man with a previous medical history of COPD and chronic liver disease was admitted to the emergency department for the evaluation of coma. Upon admission the patient was unresponsive. His blood pressure was 80/60, heart rate was 45 bpm, and body temperature was 33ºC. Laboratory tests showed undetectable T4 and TSH over 100 mU/ml. His hemoglobin was 7.8 g/dl, total cholesterol 203 mg/dl, and LDL 101 mg/dl. Electrolytes, renal, and liver tests were within the normal range. The patient received IV thyroxine and hydrocortisone. On the third day of admission, the patient was clinically improved and repeat laboratory tests showed T4 1.28 ng/dl and TSH 48.9 mU/ml.

## Discussion

Myxedema coma is a rare condition with high morbidity. This disease may be easily misdiagnosed even in patients presenting with cardinal sings such as hypothermia. The condition is more often seen in females and tends to occur during the winter period as seen in the present cases reported (December to February) [4]. Hyponatremia, hypercholesterolemia, liver or cardiac enzyme elevations, and respiratory insufficiency are frequently present. In the setting of suspected myxedema coma with increased CPK levels, free thyroxine must be assessed immediately. Some patients taking amiodarone may develop thyroid dysfunction in the form of amiodarone-induced thyrotoxicosis (AIT) or amiodarone-induced hypothyroidism (AIH) The incidence of AIH ranges from 6% in countries with low iodine intake to 13% in countries with a high dietary iodine intake [5]. Females with positive thyroglobulin or anti-thyroid peroxidase antibodies have a 13-fold higher incidence of AIH when compared to males without thyroid antibodies [3]. Measurement of T3 hormone may be helpful in cases with thyroxine hormone replacement in the setting of myxedema coma arising during treatment with amiodarone due to its extrathyroidal T3 inhibitory effect. This could explain the lack of response in our patient presenting in myxedema coma during amiodarone treatment. The diagnosis of this condition is more difficult in the elderly [3] when the T4 value is over 1 mcg/dl as seen in the euthyroid sick syndrome. In all the cases presented, we were only able to measure total thyroxine levels in the emergency department.

Treatment of myxedema coma should be immediately initiated once the diagnosis is suspected, even without laboratory confirmation. Management consists of supportive therapy, hormone replacement, and treatment of precipitating factors [1,3,4]. Intravenous thyroid hormone therapy replacement is the cornerstone in the treatment of patients. An initial intravenous dose of 300-500 μg thyroxine followed by a daily dose of 50-100 μg is preferred. Oral therapy may be started once the patient is able to tolerate oral intake Patients with thyroid hormone replacement therapy should be carefully monitored to ensure compliance and that the treatment is well-tolerated. It is also important to look for potential drug interactions (such as proton pump inhibitors, iron, or calcium supplements). With these measures, mortality related to myxedema coma in the setting of treatment noncompliance could be avoided. Myxedema coma as the initial manifestation of hypothyroidism is rarely described in the literature, given the gradual appearance of clinical symptoms suggesting the presence of a thyroid disease.

## Conclusion

Myxedema coma is a rare clinical condition associated with high mortality even when treated. The determination of thyroxine levels should be performed immediately in all patients presenting with bradycardia, bradypnea, elevated CPK, hypoxemia, and ileus.

### What is known about this topic

- Myxedema coma is a rare and serious medical condition;
- Total thyroxine or its free fraction should be determined to confirm diagnosis and before treatment onset.

### What this study adds

- Hormone replacement with T4 and T3 should be preferred in patient with previous treatment with amiodarone;
- Intravenous hormonal replacement should be preferred considering the usual presence of ileus in contrast to oral replacement described in some previous reports.

## Competing interests

The authors declare no competing interests.
